# Nicotinamide riboside prevents mitochondrial dysfunction in nemaline myopathy type 6

**DOI:** 10.1093/hmg/ddag023

**Published:** 2026-06-21

**Authors:** Rianne J Baelde, Leander A Vonk, Edgar E Nollet, Ricardo A Galli, Alexcia Fortes Monteiro, Sultan Bastu, Bornale Das, Michel van Weeghel, Bauke V Schomakers, Kasper T Vinten, Marloes van den Berg, Jolanda van der Velden, Riekelt H Houtkooper, Nicol C Voermans, Edoardo Malfatti, Coen A C Ottenheijm, Josine M de Winter

**Affiliations:** Department of Physiology, Amsterdam UMC, location VUmc, De Boelelaan 1108, Amsterdam, HZ 1081, The Netherlands; Amsterdam Cardiovascular Sciences institute, Amsterdam UMC, location VUmc, De Boelelaan 1108, Amsterdam, HZ 1081, The Netherlands; Department of Physiology, Amsterdam UMC, location VUmc, De Boelelaan 1108, Amsterdam, HZ 1081, The Netherlands; Amsterdam Cardiovascular Sciences institute, Amsterdam UMC, location VUmc, De Boelelaan 1108, Amsterdam, HZ 1081, The Netherlands; Department of Physiology, Amsterdam UMC, location VUmc, De Boelelaan 1108, Amsterdam, HZ 1081, The Netherlands; Amsterdam Cardiovascular Sciences institute, Amsterdam UMC, location VUmc, De Boelelaan 1108, Amsterdam, HZ 1081, The Netherlands; Department of Biomedical Sciences, University of Copenhagen, Blegdamsvej 3B, 2200, Copenhagen, Denmark; Department of Physiology, Amsterdam UMC, location VUmc, De Boelelaan 1108, Amsterdam, HZ 1081, The Netherlands; Amsterdam Cardiovascular Sciences institute, Amsterdam UMC, location VUmc, De Boelelaan 1108, Amsterdam, HZ 1081, The Netherlands; Department of Physiology, Amsterdam UMC, location VUmc, De Boelelaan 1108, Amsterdam, HZ 1081, The Netherlands; Amsterdam Cardiovascular Sciences institute, Amsterdam UMC, location VUmc, De Boelelaan 1108, Amsterdam, HZ 1081, The Netherlands; University Paris Est Créteil, Inserm, U955, IMRB, Créteil F-94010, France; University Paris Est Créteil, Inserm, U955, IMRB, Créteil F-94010, France; Laboratory Genetic Metabolic Diseases, Amsterdam UMC, location University of Amsterdam, Meibergdreef 9, Amsterdam AZ, 1105, The Netherlands; Core Facility Metabolomics, Amsterdam UMC, location AMC, Meibergdreef 9, 1105 AZ, Amsterdam, The Netherlands; Laboratory Genetic Metabolic Diseases, Amsterdam UMC, location University of Amsterdam, Meibergdreef 9, Amsterdam AZ, 1105, The Netherlands; Core Facility Metabolomics, Amsterdam UMC, location AMC, Meibergdreef 9, 1105 AZ, Amsterdam, The Netherlands; Laboratory Genetic Metabolic Diseases, Amsterdam UMC, location University of Amsterdam, Meibergdreef 9, Amsterdam AZ, 1105, The Netherlands; Amsterdam Gastroenterology Endocrinology and Metabolism institute, location AMC, Meibergdreef 9, 1105 AZ, Amsterdam, The Netherlands; Department of Cellular and Molecular Medicine, University of Arizona, 1656 E Mabel street, 85724, Tucson, United States; Department of Physiology, Amsterdam UMC, location VUmc, De Boelelaan 1108, Amsterdam, HZ 1081, The Netherlands; Amsterdam Cardiovascular Sciences institute, Amsterdam UMC, location VUmc, De Boelelaan 1108, Amsterdam, HZ 1081, The Netherlands; Department of Experimental Cardiology, Amsterdam UMC, location University of Amsterdam, Meibergdreef 9, Amsterdam AZ, 1105, The Netherlands; Netherlands Heart Institute, Moreelsepark 1, 3511 EP, Utrecht, The Netherlands; Amsterdam Cardiovascular Sciences institute, Amsterdam UMC, location VUmc, De Boelelaan 1108, Amsterdam, HZ 1081, The Netherlands; Laboratory Genetic Metabolic Diseases, Amsterdam UMC, location University of Amsterdam, Meibergdreef 9, Amsterdam AZ, 1105, The Netherlands; Amsterdam Gastroenterology Endocrinology and Metabolism institute, location AMC, Meibergdreef 9, 1105 AZ, Amsterdam, The Netherlands; Emma Center for Personalized Medicine, Amsterdam UMC, location AMC, Meibergdreef 9, 1105 AZ, Amsterdam, The Netherlands; Department of Neurology, Donders Institute for Brain, Cognition and Behavior, Radboud University Medical Center, Erasmusplein 1, 6525 HT, Nijmegen, The Netherlands; University Paris Est Créteil, Inserm, U955, IMRB, Créteil F-94010, France; Reference Center for Neuromuscular Disorders, APHP Henri Mondor University Hospital, Créteil, 1 Rue Gustave Eiffel, 94000, France; Department of Physiology, Amsterdam UMC, location VUmc, De Boelelaan 1108, Amsterdam, HZ 1081, The Netherlands; Amsterdam Cardiovascular Sciences institute, Amsterdam UMC, location VUmc, De Boelelaan 1108, Amsterdam, HZ 1081, The Netherlands; Department of Cellular and Molecular Medicine, University of Arizona, 1656 E Mabel street, 85724, Tucson, United States; Department of Physiology, Amsterdam UMC, location VUmc, De Boelelaan 1108, Amsterdam, HZ 1081, The Netherlands; Amsterdam Cardiovascular Sciences institute, Amsterdam UMC, location VUmc, De Boelelaan 1108, Amsterdam, HZ 1081, The Netherlands

**Keywords:** Congenital myopathy, Nemaline myopathy, Skeletal muscle, Mitochondria, NAD^+^ metabolism

## Abstract

Nemaline Myopathy type 6 (NEM6) is a congenital myopathy caused by variants in Kelch-repeat-and-BTB-(POZ)-Domain-Containing-13 (*KBTBD13*). The majority of the NEM6 patients harbor the Dutch founding variant *KBTBD13^R408**C**^* (c.1222C > T, p.Arg408Cys) and experience skeletal muscle weakness and sarcomere-based hypercontractility. Histological characterization of NEM6 patient biopsies by NADH staining shows the presence of cores, suggesting mitochondrial dysfunction. We aimed to elucidate the role of mitochondrial dysfunction in NEM6 pathology and tested the ability of the NAD^+^ precursor nicotinamide riboside (NR) to improve mitochondrial performance. We performed a natural history study in homozygous *Kbtbd13^R408C^*-knockin mice (NEM6 mouse model) to investigate the onset and progression of mitochondrial dysfunction in NEM6. We performed high-resolution respirometry, metabolic treadmill experiments and histoenzymatic NADH and SDH stainings on cryosections. Additionally, we used multi-omics analyses to investigate impacted pathways and metabolite dysregulation and performed NR supplementation for eight weeks to prevent the onset of mitochondrial dysfunction in NEM6 mice. Throughout disease progression, NEM6 mice display decreased mitochondrial respiration, impaired metabolic performance and the presence of cores with histoenzymatic reactions. Multi-omics studies revealed that the TCA cycle is heavily impacted and that NAD^+^ levels are decreased throughout disease progression. We aimed to restore NAD^+^ levels by supplementation of NR. Remarkably, NR treatment in 1-months-old NEM6 mice, prevented the onset of mitochondrial dysfunction. In conclusion, these results provide insight in the onset and progression of mitochondrial dysfunction in NEM6 and offer proof-of-concept for NR as a therapeutic strategy.

## Graphical Abstract

**Graphical Abstract:**
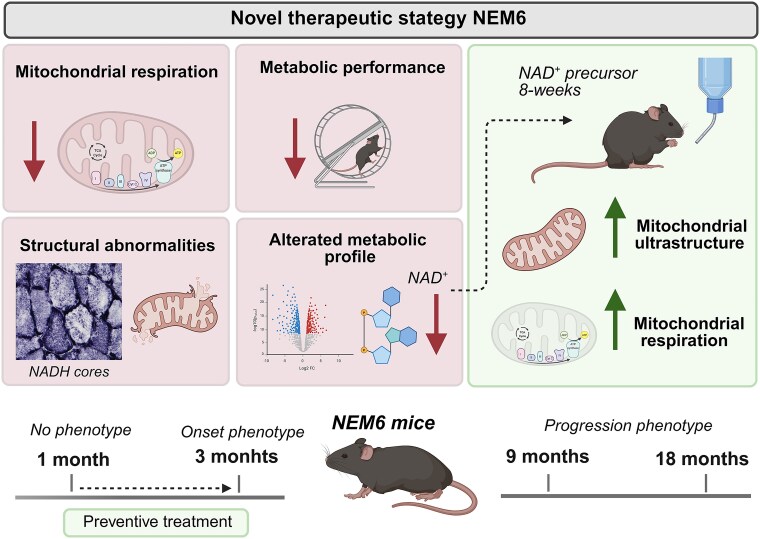

## Introduction

Congenital myopathies (CMs) are a heterogeneous group of inherited neuromuscular diseases mainly characterized by morphological abnormalities such as nemaline rods (nemaline myopathy; NEM), cores (central core and multi-minicore disease) and central nuclei (centronuclear-myotubular myopathy). NEMs are amongst the most common forms of non-dystrophic CMs [1, 2]. To date, variants in thirteen encoding proteins of the sarcomere thin filament have been associated with NEM [3, 4]. Nemaline myopathy type 6 (NEM6) is caused by variants in the *KBTBD13* gene, encoding Kelch-repeat-and-BTB-(POZ)-Domain-Containing-13 (KBTBD13), an actin-binding protein [5, 6]. The majority of NEM6 patients harbor the autosomal dominant Dutch founder variant *KBTBD13^R408C^* (c.1222C > T, p.Arg408Cys). NEM6 patients display traditional NEM hallmarks, such as muscle weakness and the presence of nemaline rods. However, NEM6 also presents unique hallmarks not observed in other subtypes of NEM [7]. For example, we previously showed that NEM6 patients experience sarcomere-based hypercontractility characterized by impaired muscle relaxation and increased myofilament calcium sensitivity [6]. This is of particular interest since most forms of NEMs are characterized by hypocontractility, resulting from decreased myofilament calcium sensitivity [8]. Another unique hallmark of NEM6 is the presences of cores, lacking NADH and SDH activity [7]. Cores are primarily associated with alterations in calcium homeostasis as observed in core myopathies [9]. However, NADH-negative cores also reflect the lack of mitochondria in these cores, hence suggesting underlying mitochondrial dysfunction [10]. By displaying both nemaline rods and cores in patients’ muscle fibers, NEM6 is classified as a core-rod myopathy [7, 11].

To date, the mitochondrial phenotype in NEM6 patients is solely based on the presence of cores, thus structural abnormalities. A recent study showed that NEM6 patients experienced increased fatigue compared to healthy controls [12]. Given that mitochondrial dysfunction can be an important contributor to fatigue, we aimed to investigate mitochondrial involvement in NEM6 and to identify a treatment strategy to improve mitochondrial function. To investigate the onset and progression of the mitochondrial phenotype in NEM6, we performed a natural history study using our previously established homozygous *Kbtbd13^R408C^* knock-in mouse model that phenocopies human NEM6 hallmarks (NEM6 mouse model) [6]. High-resolution respirometry and metabolic treadmill tests showed reduced mitochondrial respiration, impacted running performance and altered metabolism as the disease progressed. In addition, the presence of cores was accompanied by impacted TCA cycle metabolism and decreased NAD^+^ levels, as revealed by semi-targeted proteomics and metabolomics. Next, we aimed to prevent the onset of the mitochondrial phenotype with chronic supplementation of nicotinamide riboside (NR), a NAD^+^ precursor. Pre-clinical studies have shown beneficial effects of NAD^+^ precursors in improving metabolic performance, mitochondrial respiration and mitochondrial morphology in models for mitochondrial myopathy and heart failure [13–18]. Chronic supplementation of NR in NEM6 mice showed improved mitochondrial ultrastructure and prevented deterioration of mitochondrial respiration. Hence, this study discloses the presence of mitochondrial dysfunction in NEM6 and identifies NR as a supplement that prevents the onset of mitochondrial phenotype in NEM6.

## Results

### NEM6 mouse model reveals impaired mitochondrial respiration

**Figure 1:**
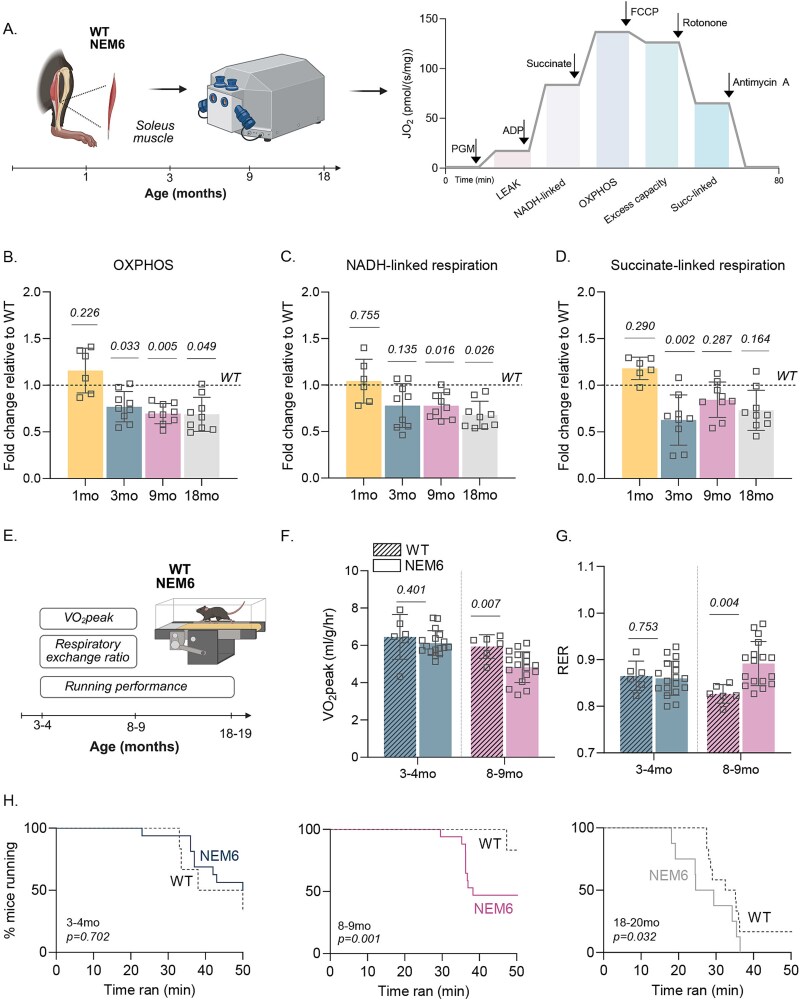
*Ex vivo* and *in vivo* functional assessment of mitochondrial phenotype in NEM6 mice. (A) Schematic overview of *ex vivo* mitochondrial function experiment using high resolution respirometry. (B-D) Total OXPHOS, NADH-linked and succinate-linked respiration in soleus muscle of 1,3,9 and 18 months old of female WT and NEM6 mice normalized to WT levels. Statistical significance was determined within age-groups by means of unpaired t-test or Mann–Whitney test for not-normally distributed data. (E) Schematic overview of *in vivo* parameters measured to determine metabolic performance. (F) VO_2_max of 3–4 and 8–9 months old male WT and NEM6 mice, measured as VO_2_ by maximum running speed. Statistical significance was determined within age-groups by means of unpaired t-test or Mann–Whitney test for not-normally distributed data. (G) Respiratory exchange ratio of 3–4 and 8–9 months old male WT and NEM6 mice measured at last time point before exhaustion or at end of running protocol. Statistical significance was determined within age-groups by means of unpaired t-test or Mann–Whitney test for not-normally distributed data. (H) Running performance of 3–4, 8–9 (n = 6 WT, n = 17 NEM6) and 18–20-months-old male mice (n = 12 WT, n = 8 NEM6) displayed as percentage of mice running throughout the 55-min running protocol. Running performance of WT and NEM6 mice was analysed by restricted mean survival time analysis (RMST) in R using area under curve and is displayed as average percentage of running time.

To study the mitochondrial involvement in NEM6 mice, we first investigated *ex vivo* mitochondrial function in WT and NEM6 littermates. *Ex vivo* mitochondrial respiration was assessed by high-resolution respirometry in oxidative soleus muscle and glycolytic EDL muscle (Fig. 1A). In 1-month-old mice, we did not observe any difference in OXPHOS in soleus muscle of WT and NEM6 mice. However, NEM6 mice revealed impaired oxidative phosphorylation capacity (OXPHOS) in soleus muscle at 3, 9 and 18 months of age (Fig. 1B). Electron transfer (ET) capacity showed a comparable trend (Supplemental Fig. 1A). Difference in OXPHOS and ET capacity (E-P excess capacity) was similar between WT and NEM6 mice throughout disease progression suggesting OXPHOS capacity in NEM6 mice is not limited by the phosphorylation system (Supplemental Fig. 1B). In addition to assessing OXPHOS and ET capacity, we also evaluated respiration on different metabolic substrates i.e. NADH-linked and succinate-linked respiration. We observed a trend towards decreased NADH-linked respiration in soleus muscle of 3-months-old NEM6 mice and a significant reduction at 9 and 18 months. For succinate-linked respiration, soleus muscle of 3-months-old NEM6 mice showed a significantly decreased respiration, but this was absent at the age of 9 and 18 months (Fig. 1C and D). We did not observe any differences in *ex vivo* mitochondrial respiration in EDL muscle (Supplemental Fig. 1C-E). To conclude, *ex vivo* mitochondrial dysfunction was absent in soleus muscle of 1-months-old NEM6 mice but starts at 3 months and continues towards 9 and 18 months.

### NEM6 disease progression results in impaired metabolic performance

To investigate whether *ex vivo* mitochondrial dysfunction impacts *in vivo* metabolic performance*,* we performed metabolic treadmill experiments in 3-4-, 8-9- and 18-20- months-old WT and NEM6 mice. We assessed the VO_2_ peak, the respiratory exchange ratio (RER) and running performance as markers for metabolic function (Fig. 1E). At 3-4-months-old, we did not find any differences in VO_2_ peak and RER between WT and NEM6 mice. These results indicate that metabolic performance was not impacted in 3-4-months-old NEM6 mice despite the impaired mitochondrial respiration at this age. However, by 8-9months we observed a significantly decreased VO_2_ peak and increased RER, indicating impaired metabolic performance and a shift in preferential substrate utilization (Fig. 1F-G, Supplemental Fig. 1F-G). In addition, we measured running performance throughout disease progression and observed impaired running performance in 8–9 (83%) and 18–20-months-old NEM6 mice (78%) (Fig. 1H). To conclude, this data reveals that NEM6-associated mitochondrial dysfunction contributes to impaired *in vivo* metabolic performance throughout disease progression.

### NEM6 mouse model recapitulates human NEM6 hallmark: The presence of cores

**Figure 2:**
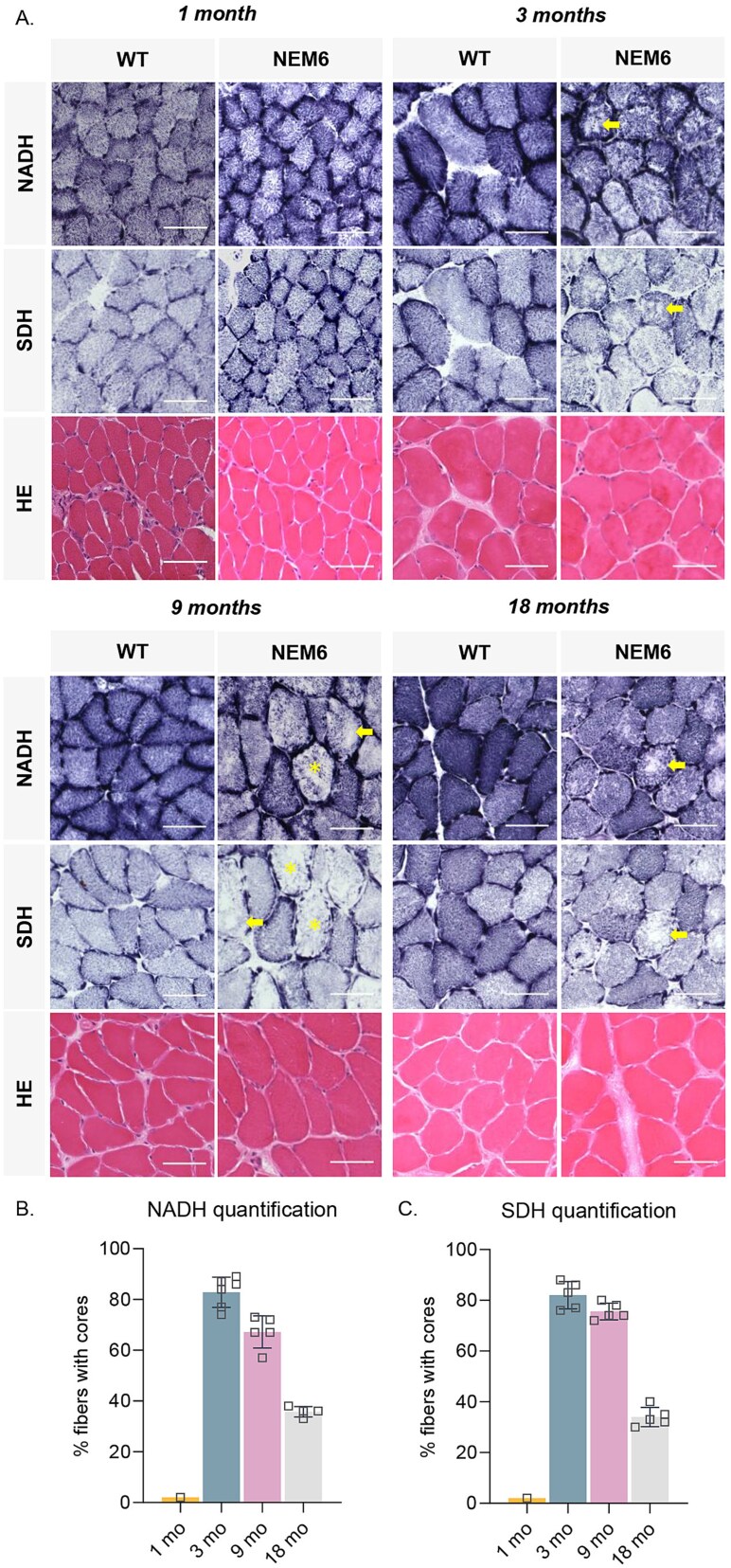
Natural history of core formation in NEM6 mice. (A) NADH, SDH and HE stainings in soleus muscle of 1, 3, 9 and 18-months-old WT and NEM6 male mice. Stainings were performed on consecutive slides. Scale bar = 50 μm. (B-C) quantification of NADH and SDH staining displayed as percentage of fibers affected by cores at 1,3,9 and 18 months old (n = 4–6 male mice).

Next, we aimed to investigate whether the NEM6 mouse model recapitulates the presence of cores observed in NEM6 patients [7]. We performed NADH and SDH histoenzymatic reactions in soleus and EDL muscle to investigate the onset and progression of cores in NEM6 mice (Fig. 2A, Supplemental Fig. 2A). We did not observe abnormal NADH and SDH activity in soleus muscle of 1-months-old NEM6 mice. However, at 3, 9 and 18 months of age, soleus muscle fibers revealed the presence of cores with both NADH and SDH histoenzymatic reaction. H&E staining appeared homogenous, indicating that muscle fiber structure is intact. Quantification of cores revealed that by the age of 3 months, the majority of the fibers contained cores when stained for NADH (83%) and SDH (82%) activity (Fig. 2B). Interestingly, 9-months-old soleus muscle showed a decreased number of fibers with cores for NADH (67%) and SDH (75%). However, in contrast to 3 months where muscle fibers harbor predominantly multiple mini cores, 15%–30% of type I fibers at 9 months showed drastically reduced NADH and SDH activity (Supplemental Fig. 2B and C). These NADH and SDH-devoid fibers are characterized by the presence of subsarcolemmal reactive areas, also known as ragged blue fibers (Fig. 2A, asterisk). At 18 months, cores were still present although the prevalence of fibers with cores was reduced with NADH (36%) and SDH (34%) reactions. It is worth noting that throughout disease progression, cores were more present in type I fibers compared to type IIa fibers (Supplemental Fig. 2B and C). In addition to the presence of cores in soleus muscle, cores were also observed in EDL muscle with a late onset at 9 months and progression towards 18 months (Supplemental Fig. 2A). In summary, we show that NEM6 mice recapitulates the formation of cores as observed in NEM6 patients. In soleus muscle, cores appear at 3 months followed by the presence of fibers devoid of histoenzymatic reaction at 9 months.

### NEM6 pathology results in altered mitochondrial morphology and abundance

**Figure 3:**
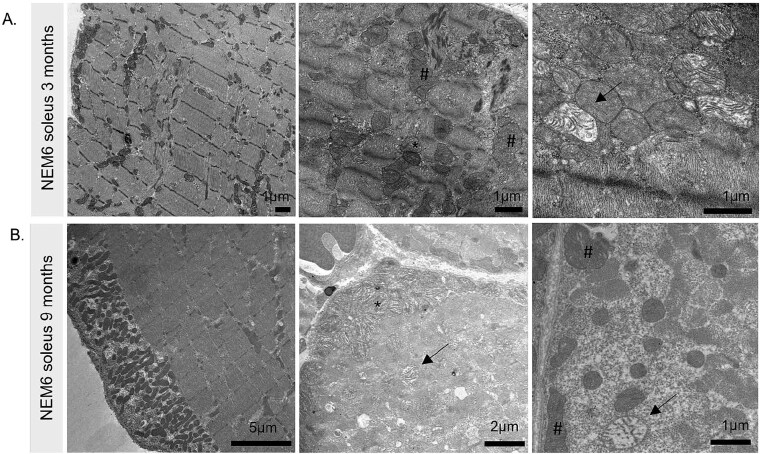
Mitochondrial morphology in NEM6 mice. EM images of soleus muscle in 3 (A) and 9-months-old (B) NEM6 mice showing abnormal mitochondrial size (#), altered cristae structure (*), severely damaged mitochondria (arrow).

Next, we performed EM to investigate whether the changes observed in mitochondrial-related function, can be explained by changes in mitochondrial morphology in NEM6 mice. EM images of soleus muscle from 3 and 9-month-old NEM6 mice show loss of densely packed mitochondrial networks (Fig. 3 left panel), abnormal mitochondrial size with the presence of swollen mitochondria and mitochondria with disordered cristae structure (Fig. 3 middle and right panel). In addition, we also observed severely damaged mitochondria, appearing white on EM images (Fig. 3, right panel). Hence, altered mitochondrial organization and structure contribute to the observed impaired mitochondrial performance in NEM6 mice.

### Multi-omics analyses reveal decreased NAD^+^ levels in NEM6 mice

**Figure 4:**
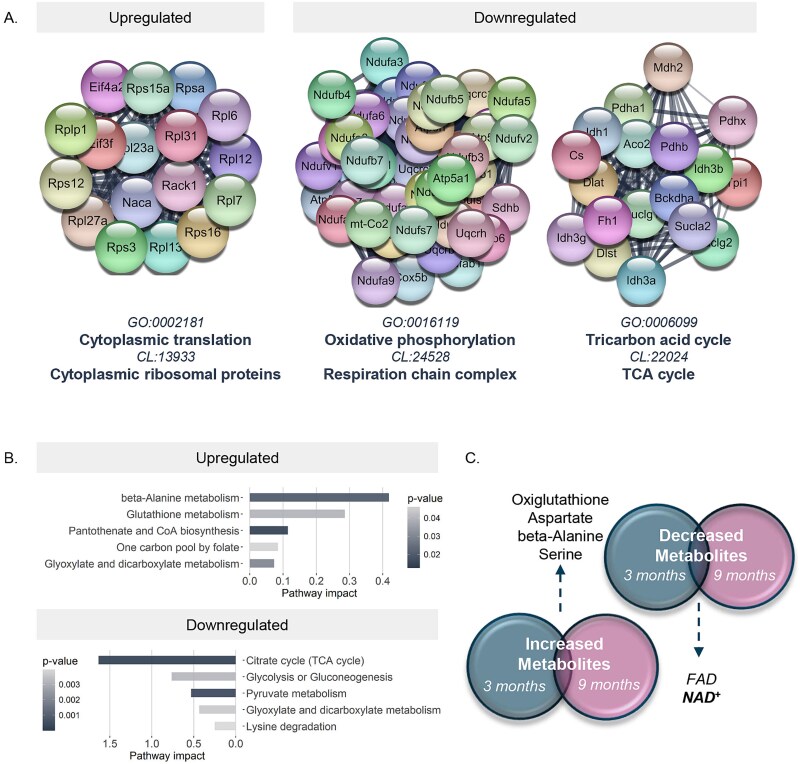
Multi-omics analyses in WT and NEM6 soleus muscle. (A). Most significant upregulated and downregulated protein cluster in 9-months-old NEM6. (B) Joint pathway analysis of proteomics and metabolic showing most significant upregulated and downregulated pathways in 9-months-old NEM6 mice. (C) Venn diagram of overlapping increased and decreased metabolites in 3 and 9-months-old NEM6 mice.

The impaired mitochondrial respiration, the presences of cores and the ultrastructural changes in both mitochondrial structure and organization raise the question about the abundance and quality of mitochondria in NEM6 mice. Hence, we performed proteomics analysis on soleus muscle of 9-months-old WT and NEM6 mice (Supplemental Fig. 3A). Clustering analysis revealed that the most upregulated network cluster was related to cytoplasmic translation, indicating increased protein synthesis in NEM6 mice. Furthermore, clustering analysis also revealed that the two most significant downregulated network clusters were related to oxidative phosphorylation, respiration chain metabolism and TCA cycle metabolism (Fig. 4A). Together with a downregulation of individual subunits corresponding to each of the five complexes of the respiratory chain, this indicates a decreased mitochondrial abundance in NEM6 mice (Supplemental Fig. 3B). In addition to the decreased mitochondrial abundance, we also examined markers indicative for mitochondrial quality. Consistent with the findings on EM images, we observed a downregulation of proteins involved in the stabilization and formation of cristae structures (Supplemental Fig. 3C). To conclude, the proteomics analysis further supports a decreased mitochondrial abundance and quality in NEM6 mice.

Next, we aimed to investigate the pathomechanism underlying mitochondrial and metabolic dysfunction in NEM6. Therefore, we performed metabolomics analysis on 9-months-old soleus muscle from WT and NEM6 mice (Supplemental Fig. 4A and B). Joint pathway analysis of combined proteomics and metabolomics at 9 months revealed the key metabolic pathways that might contribute to the impaired mitochondrial function observed *ex vivo* and *in vivo*. The most significant downregulated pathways were related to TCA cycle metabolism (*FDR = 1.09e-09*), pyruvate metabolism (*FDR = 0,0028318)* and glycolysis (*FDR = 1,27e-02)* which all supply key substrates and metabolites for oxidative phosphorylation (Fig. 4B, Supplemental Fig. 4A and B). Downregulation of those pathways could contribute to the impaired mitochondrial respiration observed in NEM6 mice. In contrast, the most significant upregulated pathway was related to glutathione metabolism *(FDR = 0.000316*), implying a role for oxidative stress in NEM6 (Fig. 4B). This observation is further supported by a downregulation of mitochondrial-specific antioxidant enzymes (Supplemental Fig. 3E). In addition, we also performed metabolomics in 3-months-old soleus muscle to identify altered metabolites throughout disease progression (Supplemental Fig. 4C and D). We visualized the overlapping altered metabolites in 3 and 9-months-old soleus muscle of NEM6 mice and observed decreased levels of NAD^+^ throughout disease progression (Fig. 4C). NAD^+^ is synthesized trough three different pathways e.g. de novo, Preiss-Handler and salvage pathway. Metabolomics revealed decreased levels of tryptophan in NEM6 mice compared to WT, indicating defects in the de novo pathway (Supplemental Fig. 4E). Boosting NAD^+^ levels in pre-clinical studies has been a promising therapeutic strategy in restoring mitochondrial function in for example mitochondrial myopathy. To conclude, multi-omics analyses revealed severely impacted TCA cycle metabolism and decreased levels of NAD^+^ in NEM6 mice throughout disease progression.

### Chronic supplementation of nicotinamide riboside prevents the onset of mitochondrial dysfunction in NEM6

**Figure 5:**
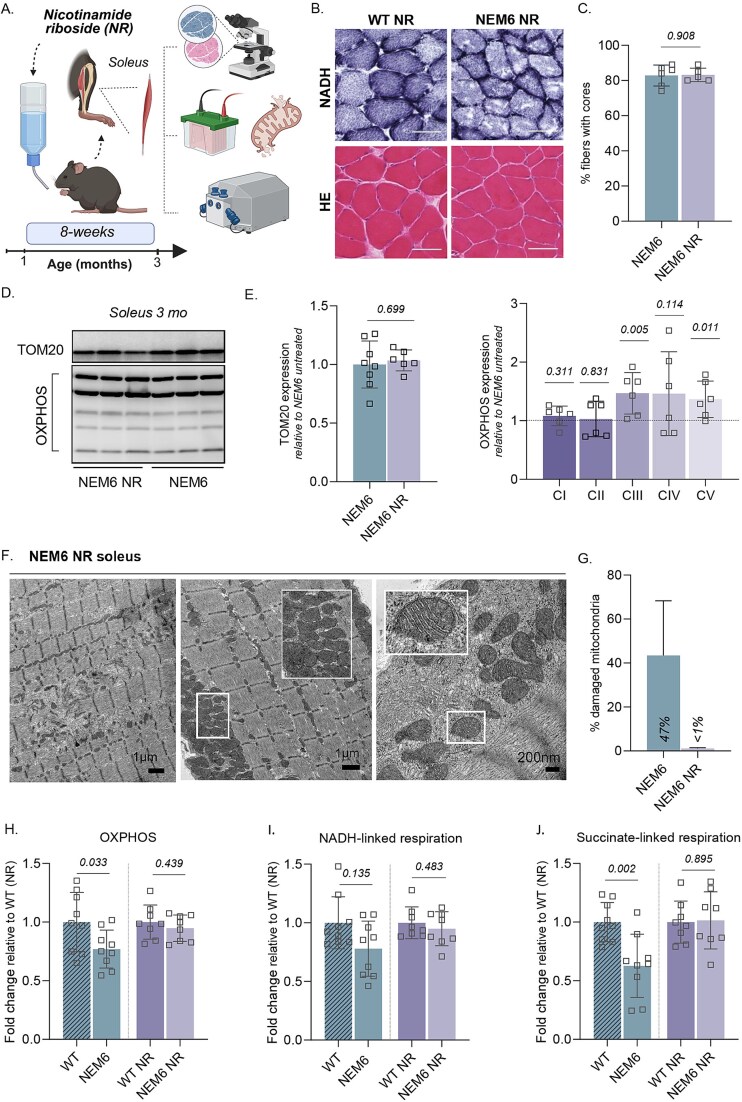
Chronic NR treatment in WT and NEM6 mice. (A) Schematic overview of 8-week NR treatment in 3-months-old WT and NEM6 mice. (B) NADH and HE stainings in soleus muscle of WT and NEM6 mice treated with NR. Stainings were performed on consecutive slides. Scalebar = 50 μm. (C) Quantification of NADH staining displayed as percentage of fibers affected by cores for untreated (n = 5 male) and NR treated NEM6 mice (n = 2 male, n = 3 female). (D) Representative western blot image of expression of TOM20 and five subunits of OXPHOS for untreated and NR treated NEM6 mice. (E) TOM20 expression and OXPHOS expression of untreated (n = 8 female) and NR treated NEM6 mice (n = 3 male, n = 3 female). Data is normalized to total protein stain and visualized as fold change compared to untreated NEM6 mice. Statistical significance was determined by unpaired t-test. (F) Representative EM images of NEM6 mice treated with NR. (G) Quantification of severely damaged mitochondria in untreated and NR treated NEM6 mice (n = 3 mice per condition). (H-J) *ex vivo* mitochondrial respiration of untreated WT and NEM6 mice and NR treated WT and NEM6 mice. Data is visualized as fold change compared to untreated WT or treated WT mice. Statistical significance was determined by unpaired t-test within treatment groups.

Based on the decreased levels of NAD^+^ throughout disease progression, we first investigated whether acute supplementation of NAD^+^ could improve *ex vivo* mitochondrial respiration in NEM6 mice. However, acute replenishing of the NAD^+^ pool did not improve mitochondrial respiration in soleus muscle of NEM6 mice (Supplemental Fig. 5A and B). Previous studies in mice showed that chronically boosting NAD^+^ levels by NAD^+^ precursors can improve mitochondrial respiration and ultrastructure [14, 19]. Therefore, we aimed to investigate the therapeutic benefit of chronically boosting NAD^+^ levels in NEM6 mice. We focused on a pre-phenotype treatment starting in 1-month-old mice, when NEM6 mice do not show any phenotype, and continued treatment up to 3 months of age (Fig. 5A). This way, we aimed to prevent the onset of mitochondrial dysfunction in NEM6 mice. To evaluate the chronic effect of NR treatment, we first determined the presence of cores. As shown in Fig. 5B and C, NR treatment did not prevent core formation in NEM6 mice (Supplemental Fig. 5C). To assess whether NR treatment resulted in a mitochondrial biogenesis response we examined the expression of TOM20 alongside five different subunits of the electron transport chain (OXPHOS) (Fig. 5D and E, Supplemental Fig. 5D). While we observe an increased expression in subunits corresponding to complex III and CV, TOM20 expression remained unaltered, indicating that NR treatment did not result in mitochondrial biogenesis. Next, we investigated whether NR treatment improved mitochondrial morphology. While disruptions of z-line continuity were still visible after NR treatment in NEM6 mice, we did observe aligned electron-dense mitochondrial networks between myofibrils. In addition, we quantified the presence of severely damaged mitochondria, cristae count per mitochondrial area and mitochondrial length as marker of mitochondrial ultrastructure. In untreated 3-months-old NEM6 mice, 47% of mitochondria are classified as severely damaged (appearing white on EM images). Interestingly, following 8-weeks of NR treatment less than 1% of mitochondria appeared to be severely damaged, reflecting improved mitochondrial ultrastructure (Fig. 5F and G). In line with that, cristae count tended to be increased *(P = 0.121)* and mitochondrial length tended to be decreased *(P = 0.103)* in NEM6 mice following 8-week NR treatment. This further supports the finding of enhanced mitochondrial ultrastructure in NR treated NEM6 mice (Supplemental Fig. 5F and G). Last, we assessed whether the improved mitochondrial ultrastructure upon NR treatment also improved *ex vivo* mitochondrial function. As shown previously, 3-months-old NEM6 mice revealed significantly decreased OXPHOS compared to WT (Fig. 1B). Most strikingly, NR treatment eliminated this difference, as OXPHOS respiration in NEM6 mice was similar to WT levels. A comparable effect was observed for NADH-linked and succinate-linked respiration (Fig. 5H-J). To conclude, NR treatment resulted in improved mitochondrial ultrastructure and prevented deterioration of mitochondrial respiration in NEM6 mice, emphasizing its potential therapeutic efficacy.

## Discussion

In this study we aimed to investigate the role of mitochondrial dysfunction in NEM6 disease onset and progression. We showed that the NEM6 mouse model recapitulates structural mitochondrial abnormalities found in NEM6 patients and provided insight into the natural history of mitochondrial involvement. Moreover, we identified NR as a potential therapeutic treatment to prevent the onset of the mitochondrial dysfunction in NEM6.

### Natural history study on mitochondrial involvement in NEM6

Thus far, mitochondrial involvement in NEM6 pathology has never been extensively studied. The mitochondrial phenotype in NEM6 patients is predominantly characterized by the presence of cores, while the contribution of *ex vivo* mitochondrial function in disease onset and progression is poorly understood [5, 7]. Hence, we performed a natural history study in a NEM6 mouse model and determined *ex vivo* an *in vivo* mitochondrial function. We established that mitochondrial dysfunction starts between 1 and 3 months, evidenced by impaired mitochondrial respiration and the presence of cores at 3 months. The mitochondrial phenotype further progresses towards 9 months as reflected by a decreased VO_2_ peak, an increased RER and impaired running performance. These *in vivo* findings may not be solely caused by mitochondrial defects, as NEM6 mice also exhibit muscle weakness from 3 months onwards, which could influence the results (Galli et al., manuscript conditionally accepted for publication). In this study we show that mitochondrial dysfunction plays a significant role in NEM6 disease progression, and likely contributes to the increased fatigue experienced by NEM6 patients [12].

### Core formation in NEM6 over time

The presence of cores is one of the hallmarks in muscle of NEM6 patients. Our NEM6 mouse model resembles this hallmark and displayed cores at 3, 9 and 18 months of age. Where muscles of NEM6 mice mainly present multi-mini-cores, central cores were most prevalent in muscle biopsies of NEM6 patients [5, 7]. However, the structure of cores in NEM6 mice and patients also share similarities. In some cases, fibers of NEM6 patients revealed regions lacking enzymatic activity centrally, while subsarcolemmal regions showed elevated enzymatic activity [7]. In soleus muscle of 9-months-old NEM6 mice, we identified similar fibers, devoid of central enzymatic activity. The presence of fibers devoid of central enzymatic activity at 9 months, compared to an absence or lower prevalence of these fibers at 3 months, indicates that the structure of cores can changes throughout disease progression. A previous study by Boncompagni et al., used a mouse model for malignant hyperthermia (MH) to investigate the structure of cores over time. This structure changed from the so-called presumptive and early cores with subtle mitochondrial damage, to cores that completely lacked mitochondria and presented severe fiber damage [20]. This might explain why the number of fibers affected by cores was decreased in 9-months-old NEM6 mice, while the mitochondrial function and morphology did not improve.

Throughout disease progression, type I fibers were more affected by cores than type IIa fibers. This is in line with a previous study in core myopathy patients carrying a variant in the ryanodine receptor (*RYR1*) [21]. However, the reason why oxidative type I fibers are more sensitive to the development of cores remains unclear. NEM6 mice presented cores at 3, 9 and 18 months, however the prevalence of fibers affected by cores at 18 months was reduced by half. In line with this finding, a previous study reported a *MYH7*-related core-myopathy patient in which cores disappeared over time [22]. The lack of improvement in mitochondrial respiration in 18-months-old NEM6 mice, despite a reduction in cores, suggests a complex interplay between structural and function mitochondrial abnormalities in NEM6 that remains to be understood. Taken together, we showed that our NEM6 mouse model recapitulates the presence of cores observed in NEM6 patients and provided insight in the development of the cores thought NEM6 disease progression.

### NAD^+^ precursor as a treatment strategy

Based on multi-omics analyses, we hypothesized that boosting NAD^+^ levels might be a therapeutic target in NEM6. In this study we showed that an 8-week treatment with NR can improve mitochondrial morphology and *ex vivo* mitochondrial respiration in soleus muscle of NEM6 mice. Comparable findings were reported by Cartwright et al., where 8-week NR treatment with a similar dosage resulted in increased mitochondrial respiration in soleus muscle of high fat diet-fed B6J mice [14]. In addition, Khan et al showed that a 16-week NR treatment with a similar dosage improved mitochondrial morphology in a mouse model for mitochondrial myopathy [19]. While we did observe improved *ex vivo* mitochondrial function and mitochondrial morphology, NR treatment had no effect on the prevalence of cores. This finding was unexpected and implies that *ex vivo* mitochondrial dysfunction is not directly correlated to the presence of cores. A possible explanation for the lack of improvement in the presence of cores is that NR treatment duration may have been too short to eliminate the cores.

Despite the promising results of NAD^+^ precursors in pre-clinical studies [13–19], the clinical efficacy in humans is not well established [23, 24]. As suggested in a recent review by Vinten et al., lower basal NAD^+^ levels might be crucial for observing functional effects of NAD^+^ precursor in humans [25]. This is supported by a previous study in patients with mitochondrial myopathy with lower basal NAD^+^ levels where 10-months treatment with NAD^+^ precursor niacin, improved muscle strength and induced mitochondrial biogenesis [26]. Since improved functional outcome parameters after supplementation with NAD^+^ precursors have not been observed consistently in humans, additional studies are required to evaluate the long-term effects of NR treatment in NEM6. Our future studies aim to investigate the effects of NR treatment on the reversibility of mitochondrial phenotype in NEM6 and improvement of metabolic and contractile function *in vivo.*

### Limitations and strengths

This is the first study that investigates the role of mitochondrial dysfunction in NEM6 disease onset and progression. It provides insight into the natural history of mitochondrial dysfunction in NEM6 and explores a potential therapeutic strategy to counteract mitochondrial dysfunction. A limitation in this study is the lack of placebo-controlled mice for the NR treatment study. In addition to that, we did not perform extended experiments to elucidate the mechanism of NR treatment in NEM6 due to limited sample availability. Notwithstanding these limitations, this study serves as a proof-of-concept study showing that NR supplementation can prevent the onset of mitochondrial dysfunction in NEM6.

### Clinical relevance

To date, the pathomechanism underlying NEM6 pathology is not fully elucidated, and no effective targeted inventions are available. NR is currently used in multiple pre-clinical and clinical studies and is reported well-tolerated and safe [25–27]. While we have provided proof-of-concept for the use of NR in NEM6, we hypothesize that targeting NAD^+^ levels might be also beneficial for other neuromuscular disorders associated with secondary mitochondrial dysfunction and fatigue. Indeed, a recent study revealed that a subset of *RYR1*-related core myopathy patients display systemic NAD^+^ deficiency. In addition, patient-derived myotubes exposed for 72 h with NR led to increased NAD^+^ levels and increased maximal respiration and ATP production [28]. These findings highlight that various neuromuscular disorders share underlying pathophysiological pathways. Understanding these common pathways can accelerate therapeutic applications.

## Materials and methods

### Animal experiments

Animal experiments were approved by the Guide for the Animal Care and Use Committee of the VU University Medical Center and by the Animal Care Committee of the VU University Medical Center (CCD-number AVD114002016700) conform the guidelines from European Directive 2010/63/EU. All animals were group-housed in a standard temperature (20–24°C) and humidity-controlled environment with 12 h light: 12 h dark cycle and ad libitum access to food and water. The NEM6 mouse model is generated as described previously [6]. Both male and females mice are included in this study since previous experiments did not show sex specific differences.

### Tissue collection

Mice were anesthetized under isoflurane (5% + 0.2 l/min O2 and 0.2 l/min air) before performing cervical dislocation. Skeletal muscles (soleus and extensor digitorum longus, EDL) were dissected and snap frozen in liquid nitrogen or used for mitochondrial respiration measurements.

### Nicotinamide riboside supplementation

Starting at the age of 4 weeks, WT and NEM6 mice received nicotinamide ribose (NR) supplementation (500 mg/kg/day) over the course of 8 weeks. This dosage was selected based on previous studies demonstrating the ability to increased NAD^+^ levels in mice [14, 29]. NR chloride (HY-123033A, Med Chem express) was dissolved in drinking water (3 mg/ml) and refreshed twice a week.

### Histology and immunohistochemical staining

Soleus and EDL muscles of WT NEM6 mice from different ages were sectioned at 10 μm at −20°C using a cryostat (Leica Biosystems, Germany).

### Enzymatic staining

Nicotinamide adenine dinucleotide dehydrogenase (NADH) staining was performed on cryosections by incubation in 0.5 M TRIS buffer (pH 7.6), 2.5 mM NBT (N6876 Sigma-Aldrich) and 2.2 mM NADH (N4505 Sigma-Aldrich) for 9 min at 37°C. Slides were washed respectively in 1 M HCL and water and mounted with glycine/gelatin (pH 7) mounting media. Succinate dehydrogenase (SDH) staining was performed on consecutive sections incubated in 0.1 M sodium phosphate buffer (pH 7.6), 0.6 mM NBT (N6876 Sigma-Aldrich) and 1.8 mM sodium succinate (S2378, Sigma-Aldrich) for 10 min at 37°C. Slides were washed in 1 M HCL and water respectively and mounted with glycine/gelatin (pH 7) mounting media. Keyence BZ-X microscope (20x magnification) was used for imaging.

### Hematoxylin and eosin staining

Slides were incubated in hematoxylin (Mayer’s hematoxylin, Sigma-Aldrich) for 5 min. After hematoxylin incubation, slides were washed with water for 5 min and stained with Eosin Y (Shandon, Thermo Scientific) for 2 min. For dehydration, slides were dipped twice in 100% ethanol. Last, slides were incubated in neo-clear (1.09843, Sigma Aldrich) for 10 min and mounted using DPX mounting media (06522, Sigma Aldrich). Keyence BZ-X microscope (20× magnification) was used for imaging.

### Metabolic performance experiments

To assess metabolic function, WT and NEM6 mice of 3–4, 8–9 and 18–20 months old underwent indirect calorimetry or conventional treadmill experiments (TSE Systems) with a graded exercise test. Mice were acclimatized one day prior to the treadmill experiments. Acclimation period consisted of: 0 m/min, 5 min; 6 m/min, 5 min; 9 m/min, 2 min; 12 m/min, 2 min. The graded exercise test consisted of: 0 m/min, 5 min; 10 m/min, 5 min; 11 m/min, 5 min; 12 m/min, 5 min; 13 m/min, 5 min; 14 m/min, 5 min; 15 m/min, 5 min; 20 m/min, 20 min or until exhaustion was reached. Exhaustion was defined as the point at which mice maintained continuous contact with the shock grid for 5 s or when there were 20 visits to the shock grid within a 60 s period. In 3–4 and 8–9-months-old mice, gas was collected continuously and analyzed every 5 s. PhenoMaster software (TSE Systems) recorded and calculated oxygen consumption (VO_2_) and the respiratory exchange ratio (RER) during exercise.

### High-resolution respirometry

Mitochondrial respiration in WT and NEM6 soleus and EDL muscle was measured using high-resolution respirometry (Oxygraph-2 k, Oroboros Instruments, Austria). Fresh saponin-permeabilized muscle bundles were prepared as described previously [30]. Leak respiration was addressed upon administration of sodium glutamate (10 mM), sodium malate (2 mM) and freshly prepared sodium pyruvate (5 mM). Maximal NADH-linked respiration was evaluated after addition of 5 mM ADP. Outer mitochondrial membrane integrity was checked upon administration of cytochrome C (1 mM). Maximal OXPHOS capacity was reached after addition of succinate (10 mM). Excess electron transferring capacity of complexes I-IV was addressed upon titration of FCCP (carbonyl cyanide-p-trifluoro-methoxyphenylhydrazone, 0.05 μM/step). Succinate-linked respiration was measured after blocking complex I by rotenone (0.5 μM) administration. After fully blocking mitochondrial oxygen consumption by antimycin A (2.5 μM), residual oxygen consumption was measured and used for background correction. O_2_ flux was normalized to wet weight of sample mass.

### Protein expression

Samples were homogenized in protein lysis buffer (105 mM TRIS-base, 140 mM TRIS–HCL, 4% LDS, 10% Glycerol, 0.51 mM EDTA, 0.22 mM Serva Blue, 0.175 mM Phenol Red, 100 mM DTT) and separated on a 4%–15% PAGE gel (Criterion TGX 5671084, Bioroad). A 2 h wet-transfer was performed to transfer samples to a PVDF membrane (immobilon-FL, Merck). A total protein stain was performed for normalization (REVERT 700, Licor 926–11 011). Membranes were blocked for 1 h at room temperature with 3% BSA in TBS-T and incubated overnight at 4°C with primary antibodies; TOM20 1:500 (ab186735, Abcam), OXPHOS 1:1000 (ab110413, Abcam). After washing with TBS-T, membranes were incubated with a horseradish peroxidase-conjugated secondary antibody 1:1000 (P0447, DAKO) for 1 h at room temperature. Protein expression was detected using ECL detection reagents (RPN2105, Amersham) and chemiluminescence imaging (Amersham Imager 600). Protein expression was quantified using ImageQuant software.

### Electron microscopy

Electron microscopy was performed on soleus muscle of 3 and 9 months old NEM6 mice. Tissue samples were fixated overnight in 0.1 M phosphate buffer (pH 7.4), 2% paraformaldehyde and 2.5% glutaraldehyde. After fixation, tissue samples were stored in 0.1 M phosphate buffer (pH 7.4) and 1% paraformaldehyde at 4°C. Upon usage, tissues were fixated in 0.1 M phosphate buffer (pH 7.4) with 2% osmium tetroxide for 1 h followed by 2% uranyl acetate for 1 h, with multiple washing step in between. Samples were dehydrated through a series of increasing ethanol and acetone concentrations. For embedding, samples were incubated overnight in 1:1 Epon acetone medium followed by 75% and 100% Epon for 1 h each. Polymerization was performed at 56°C for 3 days. Ultrathin sections (70 μm) were obtained and imaged with transmission electron microscopy. For quantification of percentage severely damaged mitochondria and mitochondrial length, 6 images per mouse were used to count all damaged and healthy mitochondria (n = 80–260 healthy mitochondria, n = 55–110 damaged mitochondria per mouse). For quantification of cristae number per mitochondrial area, cristae were counted in ~ 40 mitochondria in 3–4 high magnification images (4800-9300x) per mouse.

### Multi-omics analyses

Snap frozen soleus samples (5-8 mg) of WT and NEM6 mice processed by the Core Facility Metabolomics for multi-omics analysis. Using a liquid–liquid extraction, the polar metabolites, apolar metabolites and the protein pellet were separated and mass spectrometry-based multi-omics were performed as previously described [31]. The proteomics data have been deposited to the ProteomeXchange Consortium via the PRIDE partner repository with the dataset identifier PXD071917 and DOI 10.6019/PXD07191 [32]. Protein–protein interactions were analyzed using STRING database [33]. Protein networks were visualized using Cytoscape 3.10.4 and clustering was performed with Leiden clusterer algorithm [34]. Pathway enrichment analysis was performed on metabolomics and proteomics data set, using joint pathway analysis in MetabolAnalyst [35]. Proteins and metabolites with FDR < 0.05 were included for analysis.

### Statistical analyses

Statistics were performed with GraphPad Prism v10 software, unless otherwise specified. Data with two group comparison were analyzed using unpaired student-test for normally distributed data set. Nonparametric Mann–Whitney test was used for data sets who did not pass Shapiro–Wilk normality test. Data are displayed as mean ± standard deviation and considered significant with *P* < 0.05.

## Supplementary Material

HMG-2025-OA-01215_Baelde_revision_supplementary_data_ddag023

## Data Availability

The data underlying this article are available in PRIDE repository at PRIDE - PRoteomics IDEntifications Database, and can be accessed with [PXD071917].
