# Supplementary material for: Nicotinamide riboside prevents mitochondrial dysfunction in nemaline myopathy type 6
Source: Hum Mol Genet. 2026 Jun 21;35(12):ddag023. doi: 10.1093/hmg/ddag023 (PMC13283445; doi:10.1093/hmg/ddag023)
Supplement: HMG-2025-OA-01215_Baelde_revision_supplementary_data_ddag023 [file hmg-2025-oa-01215_baelde_revision_supplementary_data_ddag023.pdf]

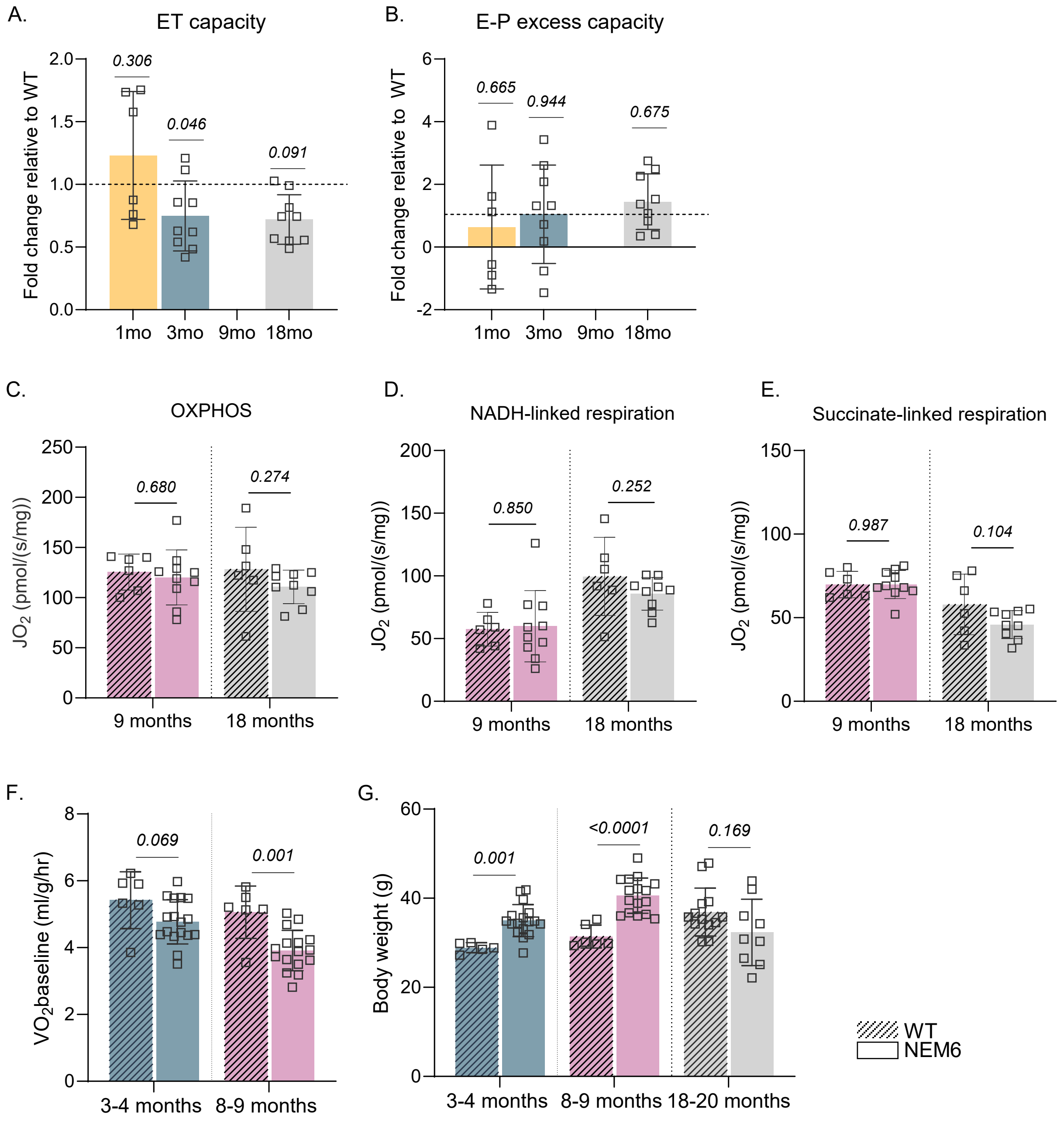

Supplementary figure 1: A) Electron transfer (ET) capacity in soleus muscle of 1,3 and 18-months-old of female NEM6 mice normalized to WT levels. Statistical significance was determined within age-groups by means of unpaired t-test. B) E-P excess capacity (ET-capacity (E) - OXPHOS respiration (P)) in soleus muscle of 1,3 and 18-months-old of female NEM6 mice normalized to WT levels. Statistical significance was determined within age-groups by means of unpaired t-test or Welch's t-test for data with unequal variance. C-E)) *Ex vivo* mitochondrial respiration (OXPHOS, NADH-linked and Succinate-linked) in EDL muscle of 9 and 18-months-old female WT and NEM6 mice. Statistical significance was determined within age-groups by means of unpaired t-test. F) VO_2 baseline of 3-4 and 8-9-months-old WT and NEM6 mice. Statistical significance was determined within age-groups by means of unpaired t-test. G) Body weight of 3-4 and 8-9 and 18-20-months-old WT and NEM6 mice. Statistical significance was determined within age-groups by means of unpaired t-test.

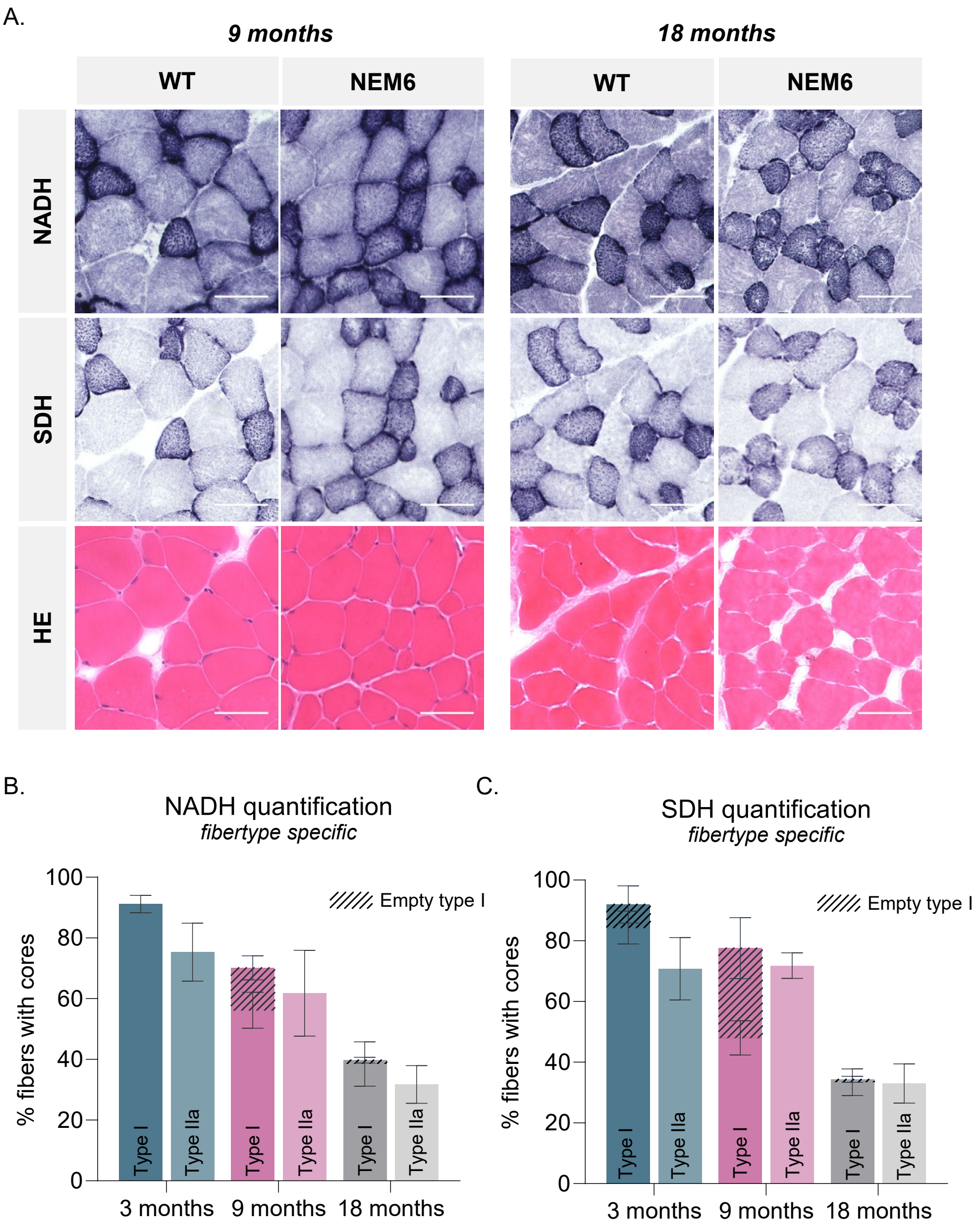

Supplementary figure 2: A) NADH, SDH and HE stainings in EDL muscle of 9 and 18-months-old male WT and NEM6 mice. Stainings were performed on consecutive slides. Scalebar=50 µm. B-C) Quantification of NADH and SDH staining in soleus muscle displayed as percentage of type I and type IIa fibers affected by cores.

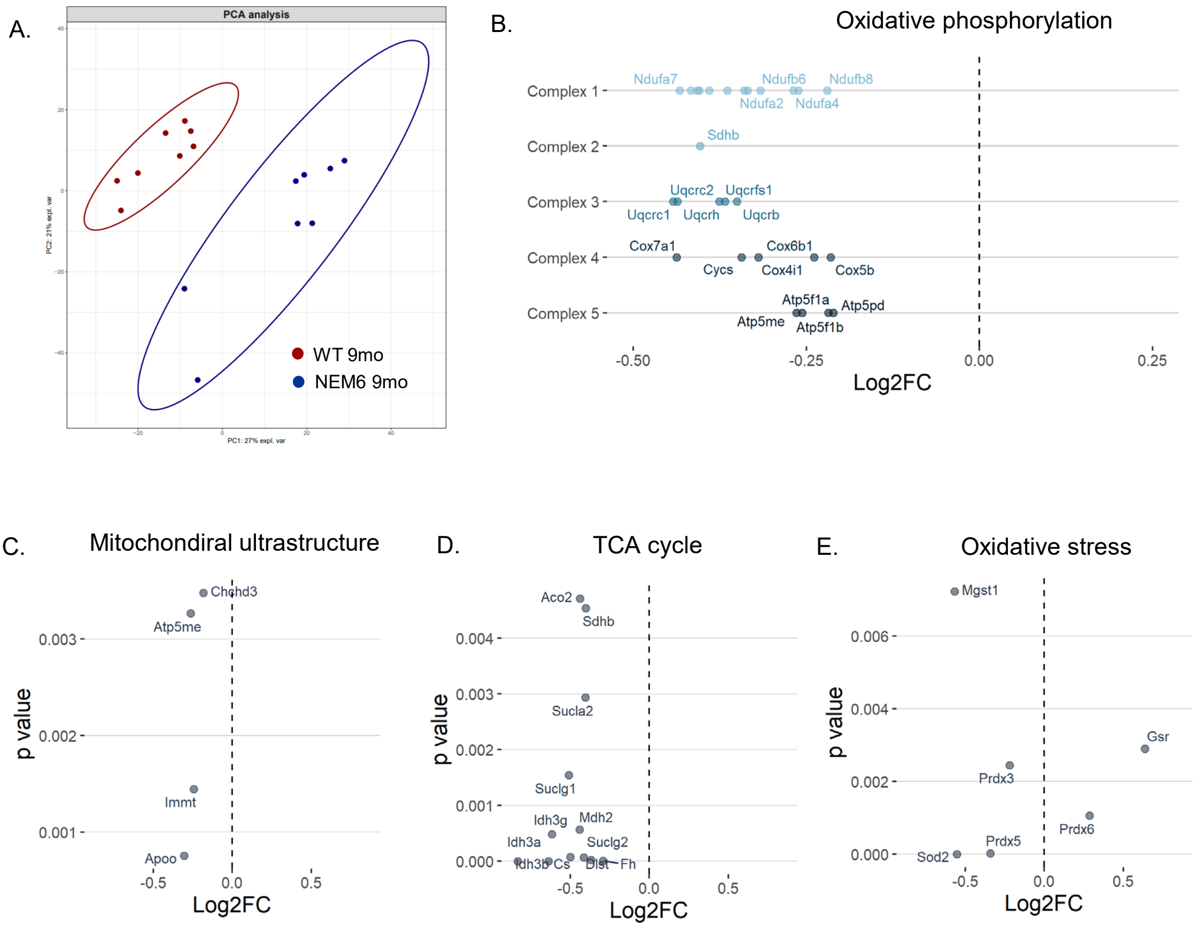

Supplementary figure 3: A) Principal component analysis (PCA) plot of proteomics in 9-monhts-old soleus muscle of male WT and NEM6 mice. B-E). Log2 fold change expression of proteins corresponding to the five subunits of electron transport chain, TCA cycle metabolism, mitochondrial ultrastructure and oxidative stress.

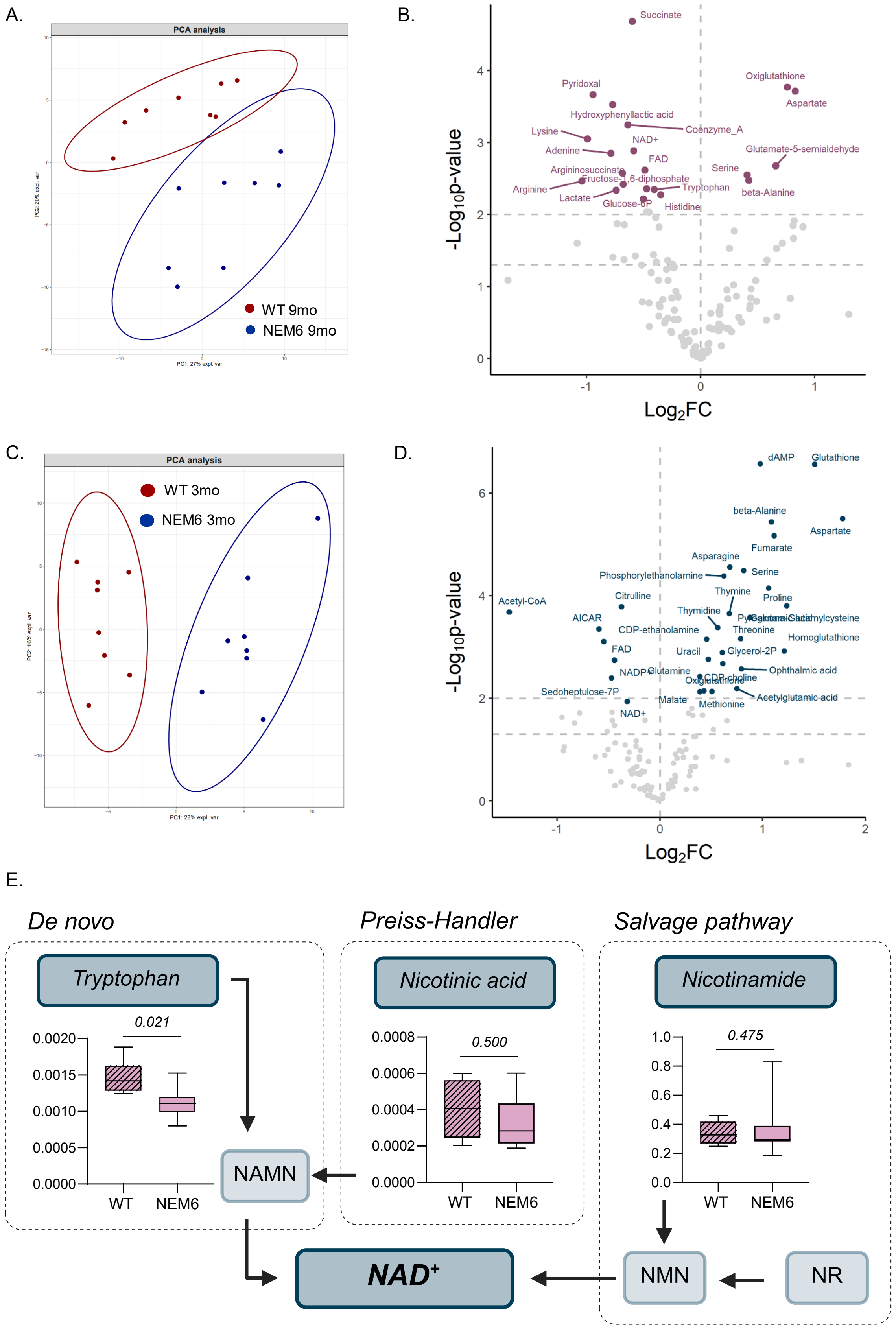

Supplementary figure 4: A) Principal component analysis (PCA) plot of metabolomics in 9-monhts-old soleus muscle of male WT and NEM6 mice. B) Volcano plot of significantly upregulated and downregulated metabolites in 9-months-old soleus muscle of NEM6 mice. C) Principal component analysis (PCA) plot of metabolomics in 3-monhts-old soleus muscle of male WT and NEM6 mice. D) Volcano plot of significantly upregulated and downregulated metabolites in 3-months-old soleus muscle of NEM6 mice. E) Schematic overview of three NAD^+ biosynthetic pathways (de novo, Preiss-Handler and salvage pathway) including tryptophan, nicotinic acid and nicotinamide levels in 9-months-old soleus muscle of WT and NEM6 mice with corresponding FDR values. NAMN= nicotinic acid mononucleotide, NMN= nicotinamide mononucleotide, NR= nicotinamide riboside.

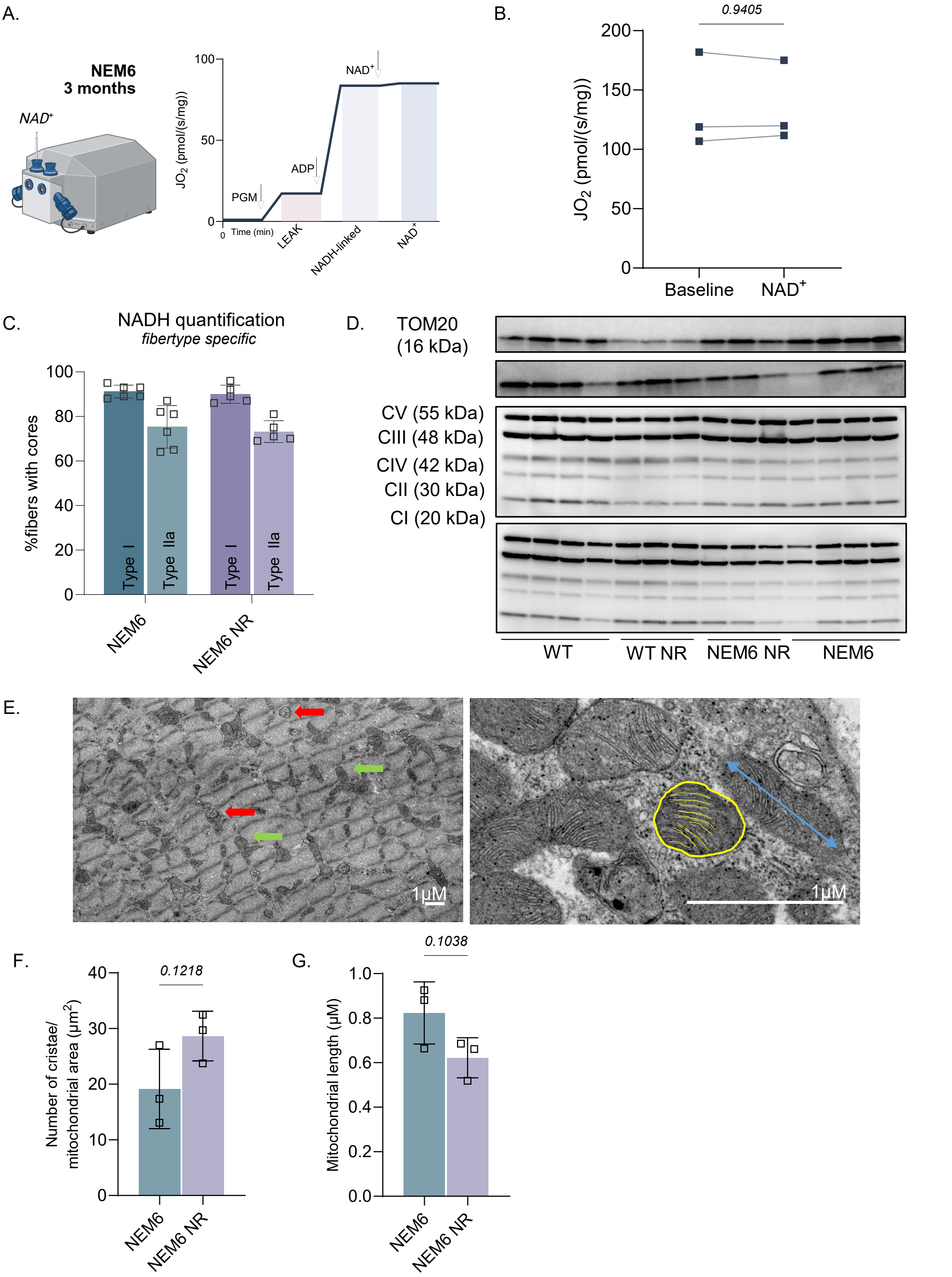

Supplementary figure 5: A) Schematic overview of *ex vivo* mitochondrial respiration experiment with administration of NAD^+. B) NADH-linked respiration before and after addition of 10μM NAD^+ in soleus muscle of 3-months-old NEM6 mice. Statistical significance was determined with paired t-test. C) Quantification of NADH staining in soleus muscle of untreated and NR treated NEM6 mice displayed as percentage of type I and type IIa fibers affected by cores. D) Western blots of TOM20 and OXPHOS subunits expression in soleus muscle of untreated and NR treated WT and NEM6 mice. E) EM quantification methods: Severely damaged mitochondrial (red arrows) and healthy mitochondrial (green arrow), cristae count normalized to mitochondrial area (yellow) and mitochondrial length (blue arrow). F) Cristae count normalized to mitochondrial area in soleus muscle of untreated and NR treated NEM6 mice. G) Mitochondrial length in soleus muscle of untreated and NR treated NEM6 mice. Statistical significance was determined with unpaired t-test.
